# Statistical Modeling of the Default Mode Brain Network Reveals a Segregated Highway Structure

**DOI:** 10.1038/s41598-017-09896-6

**Published:** 2017-09-15

**Authors:** Paul E. Stillman, James D. Wilson, Matthew J. Denny, Bruce A. Desmarais, Shankar Bhamidi, Skyler J. Cranmer, Zhong-Lin Lu

**Affiliations:** 1 The Ohio State University, Department of Psychology, Columbus, OH 43210 USA; 2 University of San Francisco, Department of Mathematics and Statistics, San Francisco, CA 94117 USA; 3 The Pennsylvania State University, Department of Political Science, University Park, PA, 16802 USA; 4 University of North Carolina at Chapel Hill, Department of Statistics and Operations Research, Chapel Hill, NC 27599 USA; 5 The Ohio State University, Department of Political Science, Columbus, OH 43210 USA

## Abstract

We investigate the functional organization of the Default Mode Network (DMN) – an important subnetwork within the brain associated with a wide range of higher-order cognitive functions. While past work has shown the whole-brain network of functional connectivity follows small-world organizational principles, subnetwork structure is less well understood. Current statistical tools, however, are not suited to quantifying the operating characteristics of functional networks as they often require threshold censoring of information and do not allow for inferential testing of the role that local processes play in determining network structure. Here, we develop the correlation Generalized Exponential Random Graph Model (cGERGM) – a statistical network model that uses local processes to capture the emergent structural properties of correlation networks without loss of information. Examining the DMN with the cGERGM, we show that, rather than demonstrating small-world properties, the DMN appears to be organized according to principles of a segregated highway – suggesting it is optimized for function-specific coordination between brain regions as opposed to information integration across the DMN. We further validate our findings through assessing the power and accuracy of the cGERGM on a testbed of simulated networks representing various commonly observed brain architectures.

## Introduction

An important and powerful approach to modeling the brain originates from network science, which views the brain as a network of interrelated regions. Network models of the brain have led to important findings about how the functional and structural organization of the brain gives rise to cognition^1–6^. Recent research finds that the whole-brain network follows small-world architecture – relatively few nodes serve as hubs that facilitate information transfer between tightly integrated communities – which in turn facilitates the integration of information across disparate regions of the brain^7–11^. The network of the brain is further organized into a series of subnetworks^12–15^ – collections of regions that are highly interrelated to contribute to a specialized set of functions.

The operating principles of these subnetworks, however, are less well understood. We investigate the operating characteristics of one such functional subnetwork active during rest as well as higher order cognitive tasks – the Default Mode Network (DMN)^16–19^. A fundamental challenge in this analysis, however, is that current statistical techniques are not suited to quantifying the operating characteristics of correlation networks (the most common way of quantifying functional connectivity between regions of the brain). Contemporary methods often require threshold dichotomization of information about connectivity and do not allow for inferential testing of the role that local processes (e.g., preferential attachment, clustering) play in determining network structure.

To address these issues, we develop the correlation Generalized Exponential Random Graph Model (cGERGM) – a statistical network model that uses local processes to capture the emergent structural (henceforth “topological”) properties of co-activation correlation networks of the brain (henceforth “functional networks”) without any loss of information. We show that the cGERGM both produces excellent fit to real brain data, and simulates realistic functional networks, making it an ideal modeling tool for quantifying functional network structure. Importantly, the parameter estimates produced by the cGERGM can be used to formally test the significance of the role of local features of the brain. Thus, the model provides a statistical method to identify fundamental properties of the network of interest. Examination of the DMN with the cGERM reveals that, rather than demonstrating small-world properties similar to the whole-brain network, the DMN appears to be organized according to principles of a segregated highway – suggesting it is optimized for function-specific coordination between brain regions as opposed to information integration across the DMN.

## Network Neuroscience

A network model of the brain typically treats regions of the brain as a collection of nodes or vertices. Edges are placed between pairs of interacting regions, and weights are placed on these edges to quantify the strength of the relationship between the two regions^6,20^. In the case of functional connectivity (the focus of the current paper), edge weights are most commonly the correlation of the time-series activation data between regions^21^ while undergoing an fMRI scan^22^. Researchers can then use statistical techniques for the analysis of networks to study the organization of the brain. The shift to studying the interrelational aspect of brain activity has produced many advances in the understanding of neuroscience, including neural mechanisms of cognitive processes such as learning and working memory^23,24^, emotion^25^, cognitive control^26^, as well as neurological diseases such as schizophrenia^27–29^.

Research also finds that the brain is organized hierarchically, with many subnetworks – often specialized to particular functions – operating and interacting to give rise to cognition^12–15^. Integral to understanding the network architecture of the brain is an understanding of how these subnetworks are organized to promote their specific functions. Subnetwork structure is often assumed to follow the same organizational principles as the whole-brain (i.e., small-world structure), though comparatively less work has been done investigating subnetwork structure (see ref.^18^). One of the primary findings in the interdisciplinary field of network science, however, is that different architectures support different functions^30–33^. Since the more specialized functions of individual subnetworks may be distinct from whole-brain synthesis and integration of information, we may expect to find different architecture for these subnetworks compared to the whole-brain. Indeed, there are many possible alternative organizations these subnetworks could display (e.g., physical proximity, random, etc.). These different network structures display different organizational properties, and in the present analysis we investigate the topological organization of one important subnetwork – the Default Mode Network (DMN). The DMN is a highly interconnected subnetwork that, in addition to being active at rest, is believed to underlie such higher-order processes as prospection^34–36^ and perspective taking^37^, leading some to conclude it is responsible for internally generated cognition^19,38^.

In this study, we hypothesize the existence of what we refer to as a “segregated highway” structure in the DMN. A “segregated highway” structure is defined as a topology with fewer hubs and more triadic closure than would be expected at random. The lack of hubs indicates the absence of preferential attachment and less of a focus on globally efficient communication across all nodes in the network. The higher levels of triadic closure are indicative of clustering. Importantly, in a segregated highway structure, there is no strong correspondence between connections generally (and triadic connections in particular) and the 3-dimensional Euclidean distance between nodes. These three attributes taken together describe a network with multiple functional specializations. The pathways through such a network are optimized by function, meaning that such a topology is ideally suited for coordinating multiple tasks over potentially disparate regions. Bringing this definition back to the highway analogy, we can imagine different highway systems optimized for task-specific information traffic. Such a system will be less efficient overall, but highly optimized within task, and compartmentalized against failure in other task-related sub-networks. Previous work on the analysis of highway structures in the brain have been recently explored in refs^39–41^.

A fundamental challenge, however, is appropriately modeling the complexities inherent in brain connectivity data in order to draw conclusions about the topological organization of the network of interest. This is challenging both due to the large number of continuous [−1, 1] weighted connections for which we must simultaneously account (~*n* ^2^ dependent observations), as well as the mathematical constraints on correlation networks. These features necessitate the development of a flexible and robust class of statistical models for correlation network data. We introduce the correlation Generalized Exponential Random Graph Model (cGERGM) – a statistical framework for modeling correlation networks – which we then use to analyze the DMN. We validate the power and accuracy of the cGERGM on a testbed of simulated networks representing various commonly observed brain architectures.

## The correlation Generalized Exponential Random Graph Model (cGERGM)

The cGERGM builds upon and extends the recently developed Generalized Exponential Random Graph Model (GERGM), which models unconstrained (−∞, ∞) weighted network data^42,43^. This class of models, however, was not designed to account for the mathematical constraints of correlation matrices. One major structural constraint is that correlation matrices must be positive semidefinite – meaning that their eigenvalues are non-negative^44^. Models that do not account for this constraint are of limited predictive utility as they cannot be used to simulate valid correlation matrices, and may lead to inferential false positives due to artifacts in the data^45^. We use a set of mathematical techniques that allow us to harness the flexibility of the GERGM, but apply it in the more restrictive and complex case of correlation network data. In so doing, we bridge neuroscience and network science to develop a modeling tool that allows for the characterization of functional networks based on essential features.

The cGERGM models two important aspects of an observed brain correlation network: (a) the *topological structure* of the network – the local and global features that describe the correlations among brain region activations such as the total edge weight of the network or the total number of triangles in the network; and (b) the influence of *exogenous features* on the connectivity of the brain regions – the effect of other relationships between the nodes, such as hemisphere location, physical distance, or any other node- or edge-level properties. The cGERGM thus allows theoretical testing of topological properties, as well as how properties of nodes or edges influence correlations between those regions, controlling for dependencies reflected in the topological structure.

Topological and exogenous features of the network are specified in the model by the user, and parameters for each of the features represent the influence of the features on the likelihood of a given network under that model. While accounting for these topological and exogenous features, the cGERGM furthermore maintains the positive semidefinite constraints of correlation matrices. Therefore, the cGERGM can be used to model a wide range of network interdependencies in functional correlation networks.

The cGERGM has four important advantages over existing models. First, the cGERGM takes all correlation information into account, and respects the mathematical constraints of correlation matrices. Many contemporary methods in network neuroscience require arbitrary thresholding of the correlation network to achieve a binary network (i.e., a connection either exists or does not). By including this potentially critical information, the cGERGM is positioned to more accurately capture network structure. Second, the cGERGM is flexible, allowing for user-specified topological and endogenous factors when modeling the correlation network. Third, the cGERGM captures and reproduces core structures of the network and can be used to simulate new networks given a set of parameters. In other words, it models the core topological processes that describe a network, and can be used to produce the emergent complexity seen in real brain correlation network data. Finally, the cGERGM produces both parameter estimates and standard errors for the unique influence of endogenous topological and exogenous effects (controlling for one another) on network structure, allowing for statistical testing and inference about the properties of the network.

The cGERGM thus offers two complementary advances for network neuroscience. First, it allows for quantification of the network using a (relatively) small number of parameters that can recreate the structure of the network. Second, and particularly important for understanding brain networks, the topological parameters recovered by the cGERGM can be used to differentiate between different network architectures, allowing researchers to understand the principles by which functional networks operate. For instance, small-world networks display heightened clustering (i.e., triadic closure) and hub usage (i.e., preferential attachment) relative to chance. Another such architecture is a “segregated highway” architecture, which displays heightened clustering but lower hub usage, and is highly optimized for function-specific coordination between regions, even across greater physical distances. An example of such a structure is a city transportation network. Trains, subways, bikes, cars, and pedestrians each travel along separate paths optimized for the particular mode of transportation^46^. Importantly, we can differentiate between these information processing architectures via assessing cGERGM parameters for different topological properties. As such, we can understand the operating principles at work in the network.

We investigate these issues in the context of the DMN, using the cGERGM to quantify the topological features that give rise to the network and allowing us to both recreate similar networks via simulation from a small number of parameters as well as gain valuable insight into the organizational principles of the DMN.

## Results

**Figure 1:**
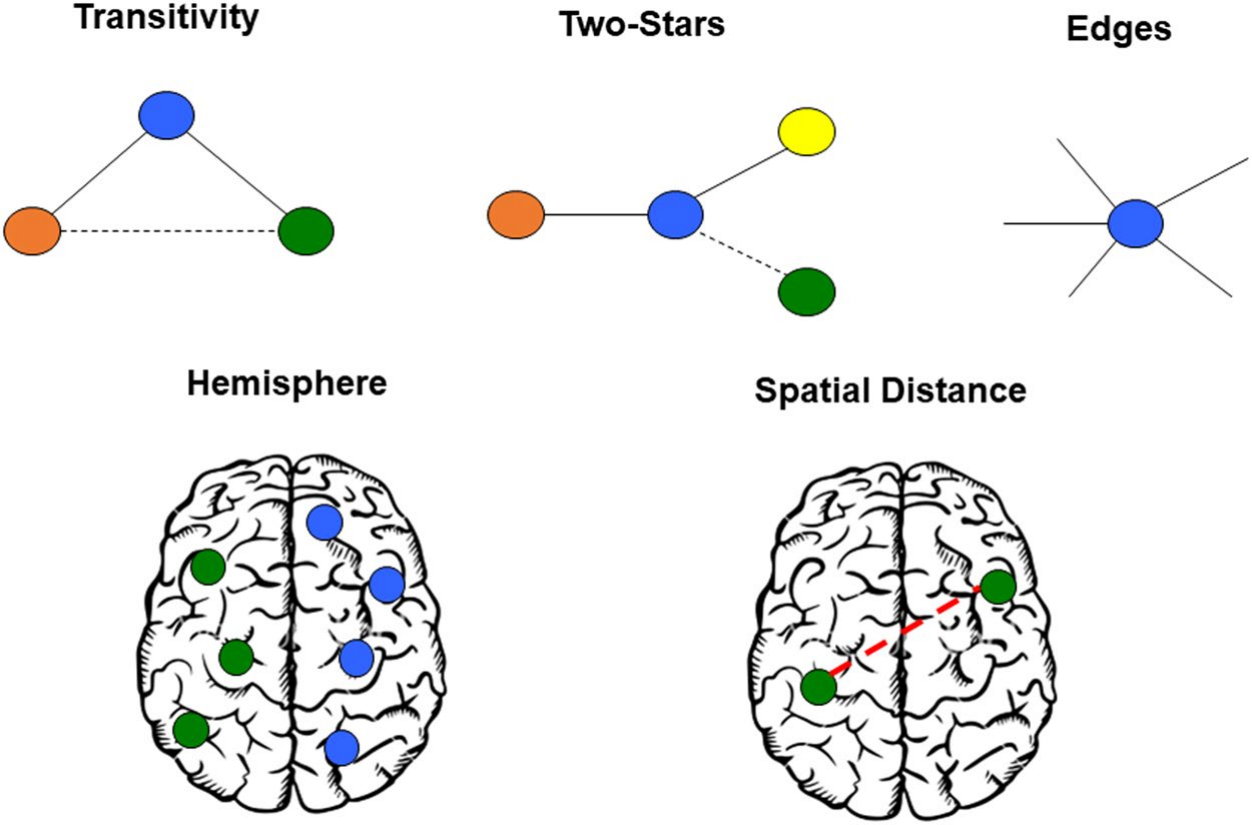
Visual depiction of each feature used in our cGERGM models. Spatial distance refers to the 3-dimensional Euclidean distance between two nodes.

We model functional connectivity in the DMN using the cGERGM (for full mathematical specification of the cGERGM, see the Methods). Brain regions are the *nodes* in this network, the correlations of the resting state functional connectivity data between the brain regions are the *edges*. In the present demonstration, we apply the cGERGM to a 20-node parcellation of the default mode network^18,47,48^. We take a dataset in which 154 participants underwent six minutes of resting state fMRI, then calculated the correlation of the 20 regions across all participants to produce the network to be modeled (models run on individuals, rather than the group, produced similar conclusions and are reported in the supplemental materials). Detailed scanning parameters and network construction details are given in the Methods. When fitting a cGERGM, the user specifies the topological and exogenous features of interest. For the present demonstration, we use five features and run 15 models – every model contained an edges feature (which models the mean edge value), and each of the 15 possible combinations of the remaining four features (see Methods and Fig. 1). Our models include two topological features: two-stars (capturing the degree of preferential attachment or hub use) and triads (capturing the degree of triadic closure or clustering). Our models further include two exogenous features – which model the effect of a covariate on connection strength – corresponding to the hemisphere of the brain in which the node resides, and the physical distance between nodes. We compare the fit of each of these models, and find that models that include topological features fit the observed network structure substantially better than those without.

### Model Specification and Estimation

**Figure 2:**
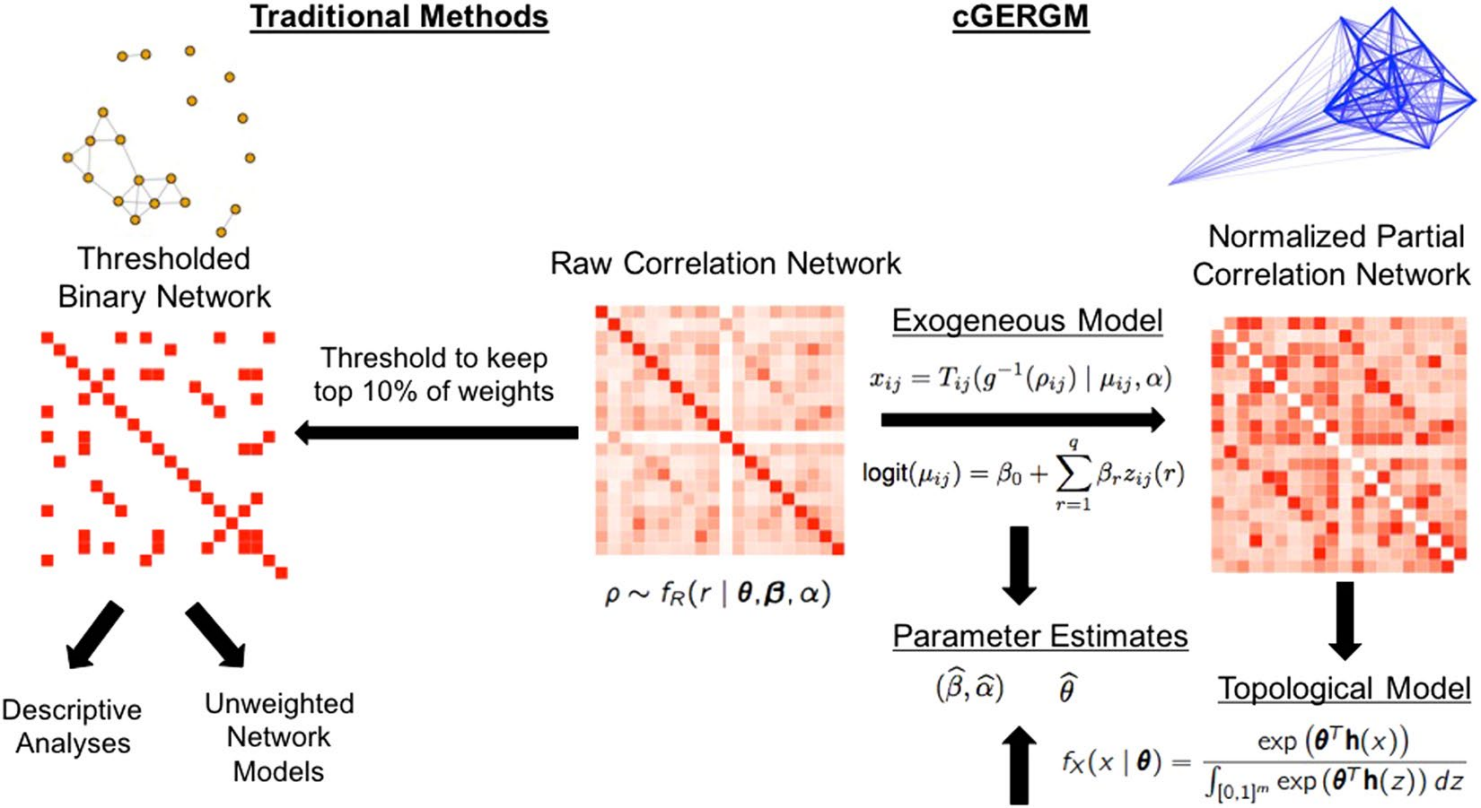
A flowchart describing the means of analyzing functional connectivity data. The left half depicts traditional methods, in which matrices are dichotomized based on some threshold (here shown at a threshold retaining the top 10% of connections), then analyzed by running descriptive statistics on the resulting thresholded network. The cGERGM approach is shown on the right, in which the network is first transformed to the constrained partial correlation space, and then topological features are assessed, in order to recover estimates of the parameters which characterize the generative process for the network under that model. Finally, these parameter estimates can be used to simulate new networks for the purpose of comparison. The images above each correlation matrix are the graphical representation for the thresholded (left) and continuous (right) networks. Note that while the graphical software does not show every edge for the weighted network case, no edges are censored to 0 for analysis purposes.

The specification of the cGERGM requires two steps (see Fig. 2 and the Methods). First, the edges of the observed correlation network, which naturally take values on [−1, 1], are transformed into normalized partial correlations that lie on [0, 1]. This transformation from correlation to partial correlation values is a key feature of the model because it allows the model to perform estimation without the positive semidefiniteness constraint, while still preserving the topological features of the network. Exogenous covariate effects are then modeled using a Beta regression of the mean of each partial correlation weight on the exogenous regressors of interest (equation (4) in the Methods). Next, the topological structure of the normalized partial correlation network is modeled using an exponential family of probability distributions that parameterize the effects of topological features on the weight of the edges within the network (equation (2) in the Methods, and see refs^42,43^).

Broadly, estimation of the parameters of the cGERGM, or any model in the (G)ERGM family proceeds as follows. First, the estimation algorithm makes an initial “guess” for all model parameters. These parameters are then used to simulate a large sample of networks from the model generative process. These networks are then used to approximate the log likelihood of the model for the current set of model parameters. The estimation algorithm then updates these parameter estimates in an attempt to simulate networks that better match the observed network, and thus yield a higher model log-likelihood. This process repeats itself until the parameter estimates converge in the sense that the change in parameter estimates at the last iteration is less than some small threshold. The final parameter estimates can then be used to perform hypothesis testing about the topological and exogenous features specified in the model. Further, it is straightforward to simulate networks from the fitted cGERGM, which are then used to assess model fit.

### Evaluating Model Fit

Before interpreting the cGERGM outputs, we first confirm adequate fit to the data. Model fit evaluation can be divided into two categories: (1) evaluating the performance of the cGERGM in making edge predictions, and (2) evaluating whether the topological features of the observed network are typical of the distributions of topological features simulated from the cGERGM.

#### Model Fit I: Edgewise Strength

**Figure 3:**
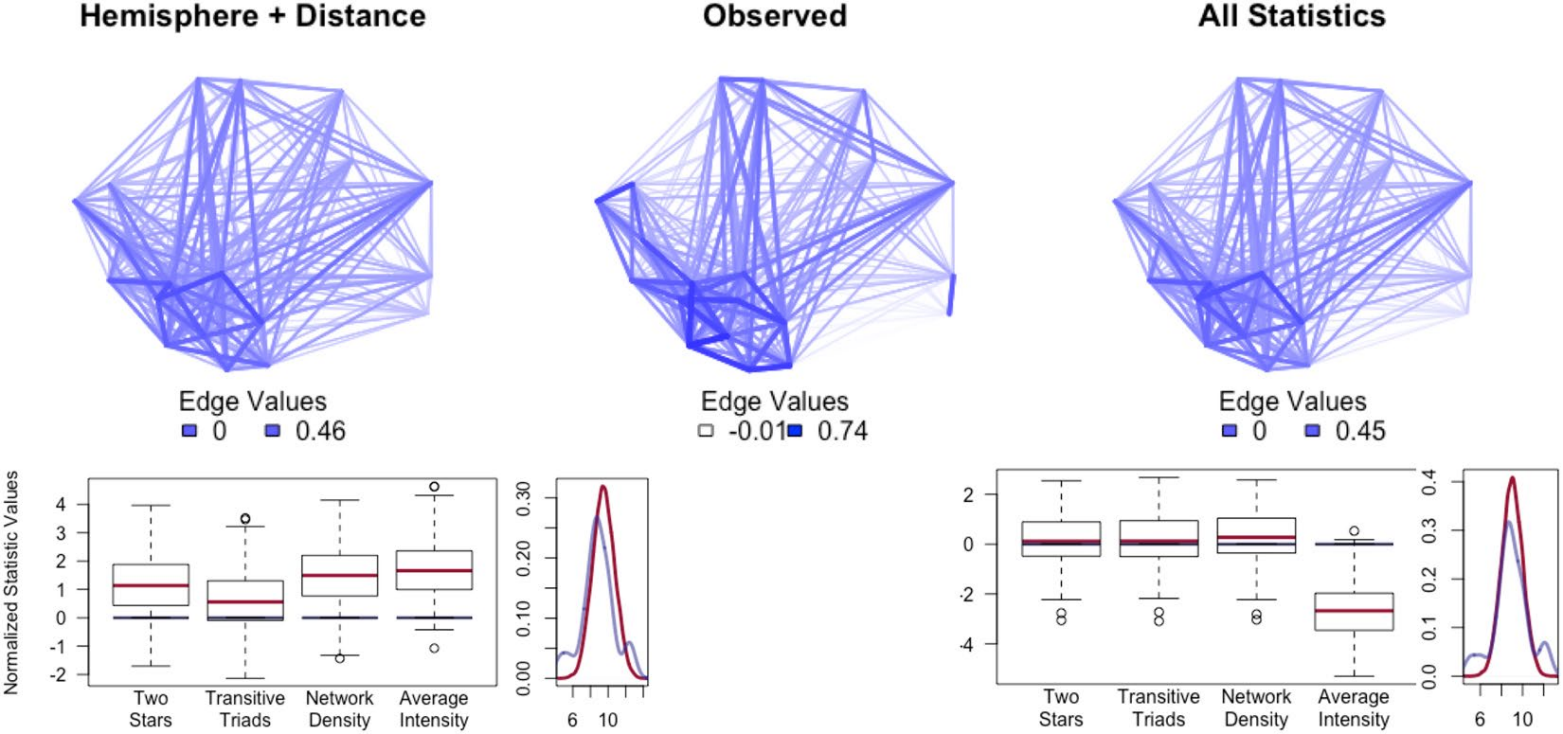
Plots illustrating both edgewise and structural fit. TOP: Observed network (middle) versus the conditional edgewise network predicted from the model with only edges, hemisphere, and distance (left) or from all features (right). From these, we can see that the conditional edgewise prediction is producing networks that closely map the observed networks. Further, the model that accounts for the topological features (right) produces more similar structure than the model which does not (left). BOTTOM: Goodness of fit for the model with only hemisphere and distance (left) versus all features (right). Boxplots reflect values from simulation based on the fitted cGERGM, with the red bars representing the mean of the simulations whereas the blue bars represent the values from the observed network. The curve to the right of each boxplot represents simulated (red) and observed (blue) degree distributions. The left model is specified to include only the edges term and the exogenous predictors of hemisphere and distance. The right model includes all topological and exogenous effects. While the model without topological features fits quite poorly, when modeling topological features our models fit quite well.

We first predict observed edge values using the fitted cGERGM. For each edge in the network, we fix all other edges at their observed value and then simulate a sample of 500 values for that edge from the fitted cGERGM. Each of these simulated edge values is used as a prediction of the given observed edge. We use these individual predictions to calculate the mean squared error (MSE) for the model predictions for each edge. When we visually compare the appearance of the observed network and these conditional edgewise models (Fig. 3), it is clear that our observed and the conditional edgewise predicted networks have strong structural similarities.

To quantify model fit across all edges, we take the average MSE from the individual edge predictions and compare this to the MSE from edges simulated using a null distribution. For this exercise, we use the maximum entropy null conditional distribution, in which we sample edge weights based only on the constraint that they maintain the positive semidefiniteness of the correlation matrix. The results of our edgewise prediction are presented in Table 1. While all models outperform the null model, there was systematic variability in model performance. We find MSEs at or below 0.012 when models included both topological features, compared to 0.024 when either topological feature is omitted. Together, this suggests both that our models recover edgewise strength beyond what would be expected by chance, and that the model has improved predictive power when topological features of the network are included.

**Table 1:** Mean squared errors for each model and the null model.

Model	MSE
Null Model	0.128
Two-Stars	0.025
Triads	0.026
Hemisphere	0.025
Distance	0.024
Two-Stars + Hemisphere	0.025
Triads + Hemisphere	0.026
Two-Stars + Distance	0.024
Triads + Distance	0.024
Two-Stars + Triads	0.012
Hemisphere + Distance	0.024
Two-Stars + Hemisphere + Distance	0.024
Triads + Hemisphere + Distance	0.024
Two-Stars + Triads + Hemisphere	0.011
Two-Stars + Triads + Distance	0.011
Two-Stars + Triads + Hemisphere + Distance	0.011

Lower numbers indicate better correspondence between observed and simulated edge values.

#### Model Fit II: Topological Features

To assess the fit of topological features of a cGERGM, we first simulate 1000 networks from the fitted model^49^. Next, topological features of the simulated networks (both those that were and were not considered in the model) are compared with the features of the observed network. We assess how well our fitted cGERGMs capture five important topological features (two of which were not included in our model specifications) in the DMN – transitive-triads, two-stars, density, degree distribution, and the intensity of the network. The degree distribution is the empirical distribution of the edge weights associated with the partial correlation network, and the intensity is the sum of the absolute deviations of each edge value from the mean edge value of the network. The bottom half of Fig. 3 illustrates the fit of two example estimated models (see Figures S2 through S6 for the results for each model fit). We see that model fit is poor when we include only exogenous features (hemisphere and distance), and that fit improves markedly when we model all features simultaneously. To statistically test the fit of a model, we calculate t-test statistics and Kolmogorov Smirnoff distances to determine whether the means of the simulated networks differ significantly from the observed. As was clear with the box-plots, when topological features were omitted from the model, there are pronounced differences between observed and simulated values for most statistics aside from the degree distribution (the *t*-values between simulated and observed networks are listed in Table 2). The average network intensity presents an interesting case—it is modeled indirectly via the scale parameter in the beta distribution, but based on Fig. 3 it looks like it would need to be included explicitly as a topological network statistic in order to be fit as well as the other features in the model. The four best-fitting models are the ones that include both topological features: two-stars and triads, as well as any combination of hemisphere and distance.

**Table 2:** Summary of model fit for each of the fitted models.

Model	Two.Stars	Triads	Edges	Intensity	Degree
Two-Stars	−1.04	**−5.68**	**6.5**	**23.5**	0.18
Triads	**26.68**	1.06	**30.86**	**37.91**	0.32
Hemisphere	**28.32**	**20.52**	**32.17**	**48.8**	0.34
Distance	**17.21**	**9.37**	**22.00**	**15.85**	0.24
Two-Stars + Hemisphere	−0.26	**−3.34**	**6.24**	**28.8**	0.16
Triads + Hemisphere	**17.9**	−1.87	**20.95**	**39.6**	0.30
Two-Stars + Distance	1.00	**−14.79**	**16.84**	**23.08**	0.17
Triads + Distance	**21.58**	0.34	**28.95**	**24.35**	0.21
Two-Stars + Triads	−1.25	−0.99	−0.98	**−39.36**	0.13
Hemisphere + Distance	**21.61**	**11.53**	**27.61**	**31.25**	0.29
Two-Stars + Hemisphere + Distance	**−2.24**	**−12.33**	**10.08**	**31.39**	0.16
Triads + Hemisphere + Distance	**25.79**	**2.00**	**33.21**	**35.65**	0.27
Two-Stars + Triads + Hemisphere	0.47	0.53	1.25	**−36.64**	0.12
Two-Stars + Triads + Distance	0.87	0.95	**3.49**	**−50.52**	0.16
Two-Stars + Triads + Hemisphere + Distance	**3.06**	**2.96**	**5.18**	**−42.68**	0.15

The two-star, triads, edges, and intensity *t* ^*^ statistic measures the normalized difference between the average mean intensity of 1000 simulated networks and the mean intensity of the observed network. The degree KS-distance is the Kolmogorov-Smirnoff distance between the empirical degree distribution of the simulated networks and the degree distribution of the observed network. Statistically significant differences in statistic values (at the *α* = 0.05 level) are displayed in bold.

### Interpreting cGERGM outputs

cGERGM results include both a parameter estimate and a standard error for each feature (parameter values produced by our models are presented in Table 3). Because interpretation of parameter estimates is only valid when the model fits adequately, we restrict interpretation to the models that include both topological features. Across all such models, we find a significantly negative two-stars parameter, and a significantly positive triads parameter. These effects are relatively unchanged with the addition of the exogenous features. Together, these results show that the DMN displays more triadic closure (i.e., clustering) and less preferential attachment (i.e., the existence of hubs) than would be expected by chance. These results are particularly notable because, contrary to hypotheses that the default mode network would display small-world structure, the observed configuration of low preferential attachment and high clustering suggest a segregated highway structure rather than a small-world structure. In other words, these results support a view of default mode connectivity that is organized around functional segregation as opposed to information transfer across the network. If some areas of the DMN served as hubs within the network, we would expect evidence of heightened preferential attachment. However, we see evidence of anti-preferential attachment, along with strong clustering, which suggests that different groups of brain regions tend to co-activate based around shared functions.

**Table 3:** cGERGM parameter estimates with standard errors in parentheses.

Model	Edges	Dispersion	Two-Stars	Triads	Hemisphere	Distance
Two-Stars	**0.27** (0.01)	**48.05** (3.43)	**−0.04** (0.01)			
Triads	**0.25** (0.01)	**47.43** (3.4)		0 (0.01)		
Hemisphere	**0.87** (0.08)	**49.23** (3.54)			**0.12** (0.03)	
Distance	**.26 (.01)**	**52.16 (3.75)**				**−0.10** (0.2)
Two-Stars + Hemisphere	**0.97** (0.08)	**50.15** (3.58)	**−0.04** (0.01)		**0.12** (0.03)	
Triads + Hemisphere	**0.89** (0.08)	**49.46** (3.55)		0 (0.01)	**0.12** (0.03)	
Two-Stars + Distance	**0.27** (0.01)	**52.80 (3.78)**	**−0.03 (0.01)**			**−0.10** (0.02)
Triads + Distance	**0.26** (0.01)	**52.30 (3.76)**		0 (0.01)		**−0.10** (0.02)
Two-Stars + Triads	**0.25** (0.01)	**38.28** (2.85)	**−0.75** (0.14)	**0.34** (0.06)		
Hemisphere + Distance	**1.00** (0.08)	**52.81** 3.80			**0.07** (0.03)	**−0.09** (0.02)
Two-Stars + Hemisphere + Distance	**1.05** (0.08)	**53.30** (3.82)	**−0.03** (0.02)		**0.07** (0.03)	**−0.08** (0.02)
Triads + Hemisphere + Distance	**1.01** (0.08)	**52.94** (3.80)		0 (0.01)	**0.07** (0.03)	**−0.09** (0.02)
Two-Stars + Triads + Hemisphere	**0.86** (0.07)	**38.95** (2.92)	**−0.85** (0.15)	**0.4** (0.07)	**0.13** (0.03)	
Two-Stars + Triads + Distance	**0.26** (0.01)	**42.85** (3.19)	**−.65** (0.12)	**0.30** (0.06)		**−0.08** (0.02)
Two-Stars + Triads + Hemisphere + Distance	**0.96** (0.07)	**42.24** (3.16)	**−0.74** (0.13)	**0.35** (0.06)	**0.09** (0.03)	**0.06** (0.02)

Effects that are statistically significant at the *α* = 0.05 level are displayed in bold.

When investigating the exogenous features, we find a significantly positive effect of hemisphere, indicating that nodes have stronger connections within the same hemisphere compared to across hemispheres. Further, we find that the Euclidean distance between nodes is significantly related to network organization, such that the further two nodes are from one another, the less strong the connection between them. This is consistent with previous research refs^50,51^ that suggests the distance between nodes is a major contributor to the organizational properties of neural networks.

## Simulation Study

To further validate our results for the application to the DMN, we assess the efficacy of the cGERGM on a testbed of simulated networks for which we know the true generative process. The aim of this simulation study is to assess the accuracy of the cGERGM on simulated networks with known parameters. We simulate networks that represent three common scenarios: (i) the simulated network displays no topological effects and therefore is a realization from a random graph (i.e., when ***θ*** = **0**), (ii) the simulated network has non-zero topological effects matching our fitted model to the DMN, and (iii) the simulated network displays significant hub structure locally. For each of these simulation studies, we simulate 1000 networks of size 20 (matching the size of the DMN) from a cGERGM specification whose coefficients represent these desired scenarios and then estimate the parameter estimates of each of the simulated networks. We report the median coefficient estimate, median standard error, and rate at which the effect was statistically significant at a 0.05 level in Table 4. Our results reveal not only that each of these common graph models can occur in a network of only 20 nodes, but also that the cGERGM accurately identifies each of the models with high power. We discuss these simulations and results below.

**Table 4:** Summary of cGERGM parameter estimates in the Simulation Study.

Statistic	Random Graph Model			Two-Stars + Triads Model			Two-Stars (Hub) Model		
	Est.	S.E.	Sign. Rate	Est.	S.E.	Sign. Rate	Est.	S.E.	Sign. Rate
Triads	−0.0253	0.1241	0.047	0.2757	0.2511	0.245			
Two-Stars	0.0317	0.1870	0.047	−0.6537	0.2543	0.684	0.478	0.033	1.00
Edges	0.5033	0.0187	1.00	0.1525	0.0945	0.429	−1.858	0.025	1.00
Dispersion	−1.5941	0.0653	1.00	0.2787	0.0732	0.908	−1.309	0.079	1.00

(Left) Networks were simulated with *θ*_*Two*−*Stars*_ = *θ*_*Triads*_ = 0 and *β*_0_ = 0.5. (Middle) Networks were simulated with *θ*_*Two*−*Stars*_ = −0.75, *θ*_*Triads*_ = 0.34 and the intercept *β*_0_ = 0.25 to reflect the Two-Stars + Triads model fitted to the DMN. (Right) Networks were simulated with *θ*_*Two*−*Stars*_ = 0.5 and the intercept *β*_0_ = −2.00 to reflect a model that demonstrates hub usage. Values are based on 1000 simulated networks and the significance rate represents the proportion of estimates that were statistically significant at a level of 0.05.

In our first study, we set *θ*_*Triads*_ = *θ*_*Two*−*Stars*_ = 0 and the intercept *β*_0_ = 0.5. We apply the cGERGM to each network with the Edges (Intercept), Two-Stars, and Triads statistics specified. The coefficient estimates from this study are consistent with the true parameter values for each simulated network. As one would hope, the two-stars and triads coefficient estimates were identified as statistically significant (non-zero) at a rate of 0.047. This result is consistent with the anticipated false discovery rate of 0.05 associated with the level 0.05 test for each coefficient. The edges and dispersion coefficients were both identified as statistically significant at a 0.05 level in all of the simulated networks. Finally, the median estimate of the intercept was 0.5053, which matches the true *β*_0_ of 0.5. This first study provides evidence of the power of the cGERGM and shows that the model does not falsely identify significant coefficients when no topological effects are present.

In the next simulation study, we assess the ability of the cGERGM to estimate the coefficients of simulated networks with non-zero topological effects. To do so, we simulate networks from an example fitted model from the DMN study provided in Table 3. In particular, we simulate networks from the fitted Two-Stars + Triads model, applying *θ*_*Two*−*Stars*_ = −0.75, *θ*_*Triads*_ = 0.34 and the intercept *β*_0_ = 0.25. We fit the cGERGM to each of the simulated networks and report the findings in the center of Table 4. We find that the estimated coefficients are consistent with the true parameter values of the simulated networks. Indeed, when considering the median estimate and standard error, we find that the true parameter values for *θ*_*Two*−*Stars*_, *θ*_*Triads*_, and *β*_0_ are all within one standard error of the median estimated value on average. Though not shown, we also calculated the number of estimations for which the cGERGM produced significant estimates that had the opposite sign from which they were generated. For both the two-stars and triads statistic, this occurred very rarely, with only one model (out of 1000) significantly positive for two-stars, and only two models (also out of 1000) significantly negative for triads. This study gives us confidence that the cGERGM is producing parameter estimates that capture the structure of the DMN.

In our last simulation, we generate networks with a strong tendency to form hubs (in other words, the presence of preferential attachment). A large amount of research has demonstrated the importance and prevalence of hubs in the context of whole-brain networks^52,53^, and it is therefore important for our model to be able to detect such preferential attachment tendencies should they exist at the subnetwork level. Notably, each of the fitted models to the DMN identified a significantly negative two-stars coefficient, suggesting a lack of hub-like structure in this subnetwork. To test whether the cGERGM can accurately identify hub structure (if it were present), we simulate networks with a significantly positive two-stars coefficient (*θ*_*Two*−*Stars*_ = 0.50) and negative edge coefficient (*β*_0_ = −2.00). The results, presented on the right of Table 4, show that the cGERGM accurately identifies both the two-stars and edges coefficients as significant in all of the simulated networks. Furthermore, the median coefficient estimates were within one standard error of the true value on average. Thus, the cGERGM can identify hub-structure in a network of the same size as the DMN.

## Discussion

We developed a novel statistical framework for quantifying brain correlation network structure – the correlation Generalized Exponential Random Graph Model (cGERGM). The cGERGM allows for the statistical modeling of both topological and exogenous predictors of correlation network structure, while simultaneously removing the need for thresholding. This allows researchers to both (a) precisely quantify the core processes underlying brain network architecture, enabling researchers to describe (and recreate) a given network using a small number of parameters, and (b) perform deductive inference on the core processes that make up a network, enabling investigation of the type of network architecture occurring in different networks. The cGERGM thus both powerfully enhances network neuroscience’s ability to quantify networks of functional connectivity, allowing researchers to both gauge the influence of different endogenous topological or exogenous features, as well as dramatically improve inference on fundamental network principles. We have shown that the cGERGM can effectively characterize the topological features of the functional default mode network, producing good fit to the observed data at both edgewise and structural levels. We further find evidence of clustering, anti-preferential attachment, hemispheric homophily, and physical distance between regions (in a way that controls for network density, transitivity, and the other parameters in the model).

We can use cGERGM outputs on topological features to infer fundamental organizational principles of the networks in question. Our results suggest that the DMN is organized by segregated highway principles wherein different functional groups of regions build their own direct channels for information sharing, rather than small-world principles where information is shared via hubs. We note that this finding may appear to run counter to previous investigations of the DMN that found evidence for hub regions in the brain^18^. This past work, however, concluded hub-behavior from significant correlations and between-network centrality. While these are properties of hubs, their presence does not guarantee that a network is characterized by preferential attachment (rich get richer) dynamics. A brain region may be highly central purely due to its position between a number of other important regions. To conclude that the DMN is characterized by preferential attachment, we need to find evidence of a rich-get-richer dynamic even when controlling for other factors such as physical proximity, as well as other processes like triadic closure which could also account for strong connections incident to a few regions. We hasten to note, however, that the present findings actually reinforce the central conclusion of this past research and others, since a segregated highway architecture strongly points towards functional segregation of the DMN^18,36^, with different regions specialized for distinct functions. Indeed, the segregated highway architecture is highly optimized for this sort of functional segregation. The present results thus demonstrate how one can quantify network properties with the cGERGM to test theoretical predictions about the structure of a given network, as well as lay the groundwork for understanding different properties of the many networks and subnetworks within the human brain.

An important caveat within these data is that, though we do not find evidence for small-world architecture, our conclusions on network activity are limited to the ways in which the regions of the DMN communicate with one another. The present results do not inform the role of these regions within the broader whole-brain network, and therefore do not run counter to past work that has found DMN regions to be hubs of the whole-brain^52,54,55^. We do note, however, that segregated highway structure appears to occur across all individuals – while there are individual differences in subject architecture, people overwhelmingly show segregated highway structure as opposed to an alternative configuration of the network (see the supplemental materials). Further work from our lab additionally finds evidence for this same architecture across different subnetworks of the brain^56^, suggesting such an architecture may be more common within the brain than originally thought. We believe the cGERGM is poised to dramatically increase the ability of network neuroscientists to systematically study the organizational properties of networks of interest.

Future work should take advantage of the powerful network quantification offered by cGERGM. For instance, because the cGERGM quantifies the unique role of each topological and endogenous feature in the network (in a way that does not require thresholding of data), the parameters recovered from cGERGM may prove more powerful and sensitive than descriptive statistics currently employed in investigations of individual or task-based differences of network architecture. Future research should investigate these possibilities through a formal comparison. Furthermore, research should also extend the cGERGM to account for longitudinal variation in correlation networks, allowing neuroscientists to harness the temporal dynamics at work in fMRI to investigate networks over the time-series^23,24^.

Future work should also continue the development and extension of the cGERGM. The present specification of the cGERGM includes only 3 structural features – edges, two-stars, and triads. We stress that the features used in the models considered here represent the beginning of possible model specifications, and future work should expand this suite of features, in particular to quantify global efficiency and rich-club coefficient – parameters past work has documented as important in cognition and disease.

Additionally, while we have focused on functional connectivity in the present paper, our approach can further be applied to neural connectivity data that is not derived from a correlation matrix. For instance, many researchers quantify structure based on white matter connectivity of the brain (e.g., via diffusion imaging) rather than correlations of activity between regions. The present statistical framework can be extended to other types of brain network connectivity data, allowing for advanced network quantification in many different contexts.

Finally, beyond neuroscience, the present method opens the topology of correlation networks to inferential study, with applications in many other domains of science. Many complex systems are such that the interaction of actors, institutions, or elements is best examined via correlation networks produced by these systems (e.g., stock prices in economics, correlations in the human genome). The cGERGM is a statistical technique that can open up these many fields to rigorous statistical analysis and quantification.

Overall, we develop and propose a new class of models for the quantification and simulation of brain correlation networks – the cGERGM. Using this, we show that the default mode network can be modeled via cGERGM parameters in a way that provides good fit. These output parameters in turn allow inferences about core principles of network structure in ways existing techniques cannot. In particular, we show here that the DMN, contrary to existing investigation of brain network organizational structure, is characterized by a segregated highway architecture, suggesting it is optimized for functionally segregated tasks.

## Methods

### Data and Network Construction

#### Use of Human Participants

All experimental protocols were approved by the Ohio State University Institutional Review Board, and all methods were carried out in accordance with relevant guidelines and regulations. All participants provided informed consent at the beginning of the study and were fully debriefed at the conclusion.

#### Participants

Two-hundred and fifty participants (147 female, ages 18–51) completed a six-minute resting state scan in which they were instructed to close their eyes and let their mind wander (but stay awake). Because excessive motion can introduce artificial correlations across regions in connectivity analyses, we opted for a strict movement cutoff whereby any participant who moved greater than 2 mm while undergoing resting state was excluded. Further, some participants had portions of their brain outside of the full field of view, and as such were excluded. Together, this yielded 154 total analyzable participants (100 female, ages 18–31).

#### Scanning parameters

Acquisition was done using a 3-T Siemens Trio scanner equipped with a 12 channel head-coil. 144 functional T2*-weighted blood-oxygen level-dependent (BOLD) echoplanar (EPI) images were acquired with TR of 2.5 s, TE of 28 ms, slice thickness of 2.5 mm, field of view = 195 * 220 mm, matrix = 78 × 88, and flip angle of 75 degrees.

#### Network construction

To construct the network, we used the functional parcellation of the DMN described by ref.^18^,supplementing this using right hemisphere coordinates^47,48^. This yielded a 20-node atlas covering both hemispheres. We then applied this atlas to each participant’s time-series data, such that each voxel within a region was averaged together, yielding 20 time-series’ for each participant. From this time-series, we partial out 6 parameters of motion, four parameters corresponding to cerebrospinal fluid, and four parameters corresponding to white matter^57^. To generate connectivity across participants, we concatenated together each subjects’ partialed time series for each ROI, and then took the Pearson correlation across all 20 ROIs. This yielded a single 20 × 20 symmetric matrix corresponding to the connection strength between each of the 20 regions. While an alternative approach would be to construct a correlation matrix for each individual participant, and then average together these matrices, we take the present strategy to conserve the properties of a correlation matrix - namely, positive semidefiniteness.

### cGERGM: a Family of Exponential Random Graph models for Correlation Networks

Our model is motivated by the need to analyze the pairwise correlations among *n* brain regions. We model this data with an undirected network represented by the weighted symmetric adjacency matrix *ρ* = (*ρ*_*ij*_: *i*, *j* ∈ 1, …, *n*). Here, we have that *ρ*_*ij*_ ∈ [−1, 1] and *ρ*_*ij*_ = *ρ*_*ji*_ for all *i* and *j*. The difficulty of directly analyzing *ρ* lies in the fact that correlation matrices must have ones along the diagonal and must be positive semidefinite, namely for any non-zero vector ***v*** of length *n*, the following relation must hold

1 $${{\boldsymbol{v}}}^{T}\rho {\boldsymbol{v}}\ge 0.$$

There are two reasons that models for correlation networks must account for the constraint of positive semidefiniteness. First, unconstrained models offer limited predictive utility as they cannot be used to simulate valid correlation matrices. Second, analyzing the structural properties of a brain correlation network without accounting for its positive semidefiniteness may lead to inferential false positives due to artifacts in the data (e.g., because they satisfy a triangle inequality, all correlation matrices exhibit non-zero transitivity relative to an unconstrained random graph^45^). For valid inference of *ρ*, we would like a model that ensures that estimates, say $\hat{\rho }$, also satisfy (1).

Our goal then is to provide a family of models for a random matrix *ρ* ∈ [−1, 1]^*n*×*n*^ that (i) upholds the positive semidefinite constraint of *ρ*, (ii) describes the relational (network) structure of *ρ*, and (iii) describes the relationship between the entries of *ρ* and a collection of potentially useful regressors *z*_1_, …, *z*_*q*_. There are some existing approaches to modeling covariance and correlation matrices (see e.g. ref.^45^); however, to the best of our knowledge no contemporary model accounts for the joint network features present in a correlation network.

In what follows, we view *ρ* as an undirected and weighted network whose edge weights (*ρ*_*ij*_: *i* < *j* ∈ [*n*]) lie in [−1, 1]^*m*^, where *m* = *n*(*n* − 1)/2 is the total number of interactions among the n regions in the brain. Briefly, our model relies on 3 transformations from the observed correlation matrix ρ to an exponential model describing the weights on the edges (see Fig. 2).

Our proposed model begins with describing the joint relational features of ρ through a constrained network *x* ∈ [0, 1]^*m*^. One first specifies a vector of features ***h***(*x*) of length *p* < *m* that describe the structural patterns of the graph x. The choice of these topological (network-structural) features is flexible, and in principle can be chosen to model any dyadic feature of a weighted graph. For instance^43^, investigated a suite of possible features for weighted graphs, and ref.^20^ described a collection of potentially useful descriptive features for brain networks. Once the features have been specified, the bounded network *x* ∈ [0, 1]^*m*^ is modeled by the joint density function

2 $${f}_{X}(x|{\boldsymbol{\theta }})=\frac{\exp ({{\boldsymbol{\theta }}}^{T}{\bf{h}}(x))}{{\int }_{{[0,1]}^{m}}\exp ({{\boldsymbol{\theta }}}^{T}{\bf{h}}(z))dz},$$

where ***θ*** is a *p* - dimensional vector of parameters that quantify the effects of the network features ***h***(*x*) on the likelihood of the graph *x*.

In the next step, we generate a vector of unconstrained partial correlations *ϕ* = (*ϕ*_*ij*_: *i* < *j* ∈ [*n*]) ∈ [−1, 1]^*m*^ by applying the transformation *x*_*ij*_ = *T*_*ij*_((*ϕ*_*ij*_ + 1)/2|*μ*_*ij*_, *α*), where *T*_*ij*_(·|*μ*_*ij*_, *α*) is the cumulative distribution function of a Beta distribution with mean *μ*_*ij*_ and scale parameter *α*. Let $T:{{\mathbb{R}}}^{m}\to {\mathrm{[0,}\mathrm{1]}}^{m}$ be defined as the m-dimensional vector *T*(*ϕ*|***μ***, *α*) = (*T*_*ij*_(*ϕ*_*ij*_|*μ*_*ij*_, *α*), *i* < *j* ∈ [*n*]). Then *ϕ* is a random vector from the probability density function *f*_Φ_(*ϕ*|***θ***, ***μ***, *α*), given by

3 $$\begin{array}{ccc}{f}_{{\rm{\Phi }}}(\varphi |{\boldsymbol{\theta }},{\boldsymbol{\mu }},\alpha ) & = & {f}_{X}(T(\frac{\varphi +1}{2})|{\boldsymbol{\theta }})\prod _{ij}{t}_{ij}(\frac{\varphi +1}{2}|{\mu }_{ij},\alpha )\\ {t}_{ij}(w|{\mu }_{ij},\alpha ) & = & \frac{{\rm{\Gamma }}(\alpha )(1-{w}_{ij}{)}^{(1-{\mu }_{ij})\alpha -1}}{{\rm{\Gamma }}({\mu }_{ij}\alpha ){\rm{\Gamma }}((1-{\mu }_{ij})\alpha )}{w}_{ij}^{{\mu }_{ij}\alpha -1},\end{array}$$

where ${\rm{\Gamma }}(t)={\int }_{0}^{\infty }{x}^{t-1}{e}^{-x}dx$ is the gamma function defined for t > 1 and *w*_*ij*_ ∈ (−1, 1). A notable property of (3) is that when the partial correlation network *ϕ* does not contain any network structure, i.e. when ***θ*** = **0**, then the m components of (*ϕ* + 1)/2 are independent samples from a Beta(*μ*_*ij*_, *α*) distribution, where *μ*_*ij*_ ∈ (0, 1) is the expected value of (*ϕ*_*ij*_ + 1)/2 and *α* is the precision of the distribution.

Model (3) provides a flexible avenue to evaluate the effect of exogeneous variables *z*_1_, …, *z*_*q*_ on the partial correlation matrix *ϕ*. Let ${z}_{\ell }$ be a vector of dyadic observations ${z}_{\ell }=({z}_{ij}(\ell ):i < j\in [n])$ for $\ell =1,\ldots ,q$. We model the effect of each predictor using a generalized linear model for the mean ***μ*** of *ϕ*:

4 $$\text{logit}({\mu }_{ij})={\beta }_{0}+\sum _{r=1}^{q}{\beta }_{r}{z}_{ij}(r)$$

Since *μ*_*ij*_ ∈ (0, 1), the logit(·) link in (4) is a natural choice, but any monotonically increasing and twice differentiable function can be used here. The regression model in (4), together with the Beta density *t*_*ij*_(·|*μ*_*ij*_, *α*) is well-studied and generally referred to as Beta regression^58^.

The final step in defining the cGERGM relies on the relationship between the partial correlation matrix *ϕ* and its associated correlation matrix *ρ* for the collection of n brain regions. Let *ϕ*_*jj*+*k*_ denote the partial correlation between region *j* and *j* + *k* given regions *j* + 1, …, *j* + *k* − 1 for *k* ≥ 2. A well-known result in multivariate statistics is that there exists a one-to-one mapping between *ρ* and *ϕ* ^59^. Given *ϕ*, one can calculate the correlation network *ρ* using the following recursion:

5 $${\rho }_{jj+k}=\{\begin{array}{cc}1 & k=0\\ {\varphi }_{jj+k} & k=1\\ {{\boldsymbol{\rho }}}_{1}^{T}(j,k){D}_{jk}^{-1}{{\boldsymbol{\rho }}}_{2}^{T}(j,k)+{\varphi }_{jj+k}{C}_{jk} & 2\le k\le n-j,\end{array}$$

where

$$\begin{array}{rcl}{{\boldsymbol{\rho }}}_{1}^{T}(j,k) & = & ({{\boldsymbol{\rho }}}_{jj+1},\ldots ,{{\boldsymbol{\rho }}}_{jj+k-1}),\\ {{\boldsymbol{\rho }}}_{2}^{T}(j,k) & = & ({{\boldsymbol{\rho }}}_{j+kj+1},\ldots ,{{\boldsymbol{\rho }}}_{j+kj+k-1}),\\ {C}_{jk}^{2} & = & [1-{{\boldsymbol{\rho }}}_{1}^{T}(j,k){D}_{jk}^{-1}{{\boldsymbol{\rho }}}_{1}^{T}(j,k)][1-{{\boldsymbol{\rho }}}_{2}^{T}(j,k){D}_{jk}^{-1}{{\boldsymbol{\rho }}}_{2}^{T}(j,k)],\end{array}$$

and

$${D}_{jk}=(\begin{array}{cccc}{\rho }_{j+1j+1} & {\rho }_{j+2j+1} & \cdots  & {\rho }_{j+k-1j+1}\\ {\rho }_{j+1j+2} & {\rho }_{j+2j+2} & \cdots  & {\rho }_{j+k-1j+2}\\ \vdots  & \vdots  & \vdots  & \vdots \\ {\rho }_{j+1j+k-1} & {\rho }_{j+2j+k-1} & \cdots  & {\rho }_{j+k-1j+k-1}\end{array})$$

Represent the multivariate transformation from *ρ* to *ϕ* given by the recursion in (5) by *ρ* = *g*(*ϕ*). Then the Jacobian of this transformation, as shown by ref.^60^,is given by

$$|J(c)|={[\prod _{i=1}^{n-1}{(1-{c}_{ii+1}^{2})}^{n-1}\prod _{k=2}^{n-2}\prod _{i=1}^{n-k}{(1-{c}_{ii+k}^{2})}^{n-k-1}]}^{-1/2}$$

Applying the inverse probability transform to the density in (3), we can generate a *positive semidefinite* correlation network *ρ* from the density:

$${f}_{R}(\rho |{\boldsymbol{\theta }},{\boldsymbol{\mu }},\alpha )={f}_{{\rm{\Phi }}}({g}^{-1}(\rho )|{\boldsymbol{\theta }},{\boldsymbol{\mu }},\alpha )|J({g}^{-1}(\rho ))|,$$

In summary, for fixed parameters ***θ*** = (*θ*_1_, …, *θ*_*p*_), ***β*** = (*β*_0_, …, *β*_*q*_), and *α* > 0 the cGERGM model for a correlation network *ρ* on n brain regions is described by the following generative process

6 $$\begin{array}{ccc}\rho  & \sim  & {f}_{R}(\cdot |{\boldsymbol{\theta }},{\boldsymbol{\beta }},\alpha )\\ \text{logit}({\mu }_{ij}) & = & {\beta }_{0}+{\beta }_{1}{z}_{ij}(1)+\ldots +{\beta }_{q}{z}_{ij}(q).\end{array}$$

### Model Specification

To fit a cGERGM to an observed correlation network, we need to specify topological features to be included in ***h***(*x*), and/or exogenous predictors for the regression function *T*(·|***β***). The corresponding vectors of coefficients, ***θ*** and ***β***, describe the effects of the specified features on the generative process of the observed weighted graph. In our investigation, we fit a family of cGERGMs to the DMN using a suite of topological and exogenous features that represent important processes in the brain. A visualization of the features that we use are available in Fig. 1. We discuss these features in more detail in the remainder of this section.

#### Topological features

We consider three topological features as predictors in our fitted models – two-stars, triads, and edges. The two-stars statistic is a measure of preferential attachment in the brain^61^. This value will be higher for networks in which there is a high variance in the popularity of vertices (i.e., those in which there exist highly connected hubs). The two-stars statistic is highly relevant to network approaches to neuroscience, as it closely associates with the extent to which hubs are present in a given network relative to pure chance. This statistic directly allows for inferential testing of the role of hubs in a given network, which we expect to be significant given the importance for hubs in brain networks^53^.

The next term we include, the triads statistic, is a measure of clustering of the regions in the DMN. In an unweighted network, this statistic counts the number of closed triangles in a network. In weighted networks, the triads statistic expresses the overall strength of connection of triangles in the restricted network *x*. As with preferential attachment, a high degree of clustering has been consistently shown to be a feature of brain networks^7,11,20^. Furthermore, reductions in clustering (in addition to other metrics), have been shown to be closely associated with disease states, such as schizophrenia^28^.

The edges term, which we also include, parameterizes the mean edge value in the restricted network. The parameter estimate for this value is analogous to an intercept term in a standard regression model, and is included as part of the transformation of exogenous covariates onto the network (described in the next section). We note that including an edges term is important for model fitting as this term normalizes the overall connectivity of the observed network so as to avoid erroneous inflation of the parameter estimates for the remaining features.

#### Exogenous features

Exogenous features are covariates that capture properties of the DMN at a node or dyad level. The parameter estimate associated with an exogenous feature describes the influence of the specified feature on the overall strength of an edge in the observed correlation network. Exogenous features can be either categorical or continuous variables.

In our study, we consider two relevant exogenous features that describe the spatial relationship of the regions in the DMN—hemisphere location and distance between regions. The hemisphere feature is a dyad-level variable that indicates whether or not a pair of nodes (regions) are located in the same hemisphere of the brain, either the left hemisphere, right hemisphere, or the midline. Given that regions within a hemisphere are typically more connected within rather than across hemisphere^62^, we expected this covariate to be significantly positive.

The second feature that we consider in our model specification is the physical distance between nodes. For each pair of regions, this feature records the physical distance between the center of each ROI in the DMN. The distance feature accounts for the functional similarity of regions that are physically close to one another in space. Past work in neural architecture has identified distance as being an important driving factor for the development of connections, with closer regions having stronger connections than further regions^8,51^. While distance certainly plays a crucial role in the architecture of connections, it is unclear whether this will be present in the default mode – a network spatially dispersed across many regions of the brain.

#### Our Fitted Network Models

Necessary inputs for estimating the cGERGM include the network data (i.e., the correlation matrix), exogenous covariate data, and the list of topological features. For the present demonstration, we fit 15 different models to the DMN. All models include an edges feature (which corresponds to an intercept), and the remaining features correspond to all 15 possible combinations of our four topological and exogenous features. We set *T*(·|*β*) as a Beta distribution, and model the partial correlations as a regression on the exogenous predictors using Beta regression^58^. To avoid model degeneracy – a common issue in exponential random graph models^63^ – we applied a geometric down-weighting strategy to the structural predictors as described by ref.^43^. This resulted in geometric downweights of 0.8 for two-stars and 0.4 for triads.

#### Data and Code Availability

For all analyses, we used the R package *GERGM* to fit the specified models. Upon acceptance for publication, all code necessary for reproduction, as well as the network data and model output, will be posted at the Open Science Framework, osf.io/d5yk7.

### Fitting the cGERGM

To fit a cGERGM (equation 4 in the main text) to an observed correlation network *ρ*, one needs to only define topological (network-level) features ***h***(*x*) = (*h*_1_(*x*), …, *h*_*p*_(*x*)) and exogenous features *z*_1_, …, *z*_*q*_. We then apply the Metropolis–Hastings Markov Chain Monte Carlo methodology from ref.^43^ to calculate the maximum likelihood estimators for the fixed unknown parameters ***θ*** and ***β***. The resulting parameter estimates $\hat{{\boldsymbol{\theta }}}$ and $\hat{{\boldsymbol{\beta }}}$ describe the effects of the topological and exogenous features on the likelihood of *ρ*, respectively. These parameter estimates can be used to further explore the nature of the data through simulation and testing.

## Electronic supplementary material

Supplementery Information
