# Supplementary material for: Statistical Modeling of the Default Mode Brain Network Reveals a Segregated Highway Structure
Source: Sci Rep. 2017 Sep 15;7:11694. doi: 10.1038/s41598-017-09896-6 (PMC5601943; doi:10.1038/s41598-017-09896-6)
Supplement: Supplementary file 1 — Supplementery Information [file 41598_2017_9896_MOESM1_ESM.pdf]

Statistical Modeling of the Default Mode Brain Network Reveals a Segregated Highway Structure
Supplemental Material

Paul E. Stillman[a], James D. Wilson[b], Matthew J. Denny[c], Bruce Desmarais[c], Shankar Bhamidi[d], Skyler Cranmer[a], Zhong-Lin Lu[a]

[a] *Ohio State University*
[b] *University of San Francisco*
[c] *Penn State University*
[d] *University of North Carolina at Chapel Hill*

Supplemental Results – models of individual subjects

In the main text, we report results of a single correlation matrix that concatenated across all subjects. We did this for two reasons, first, it allows us to achieve an estimate of the central tendency (across participants) of the correlations between regions. Second, while the estimation procedure can sometimes converge relatively quickly (e.g., approximately 20 hours) for a given connectivity matrix, in some cases the algorithm can take days or weeks to converge, making processing all 154 subjects infeasible. To address this, for each subject, we conducted an initial search, in which we allowed cGERGM to run for approximately 24 hours for each participant. If the algorithm did not converge in this time, the program continued on to the next participant. For the individual models, we use the model with all covariates (edges, two-stars, triads, hemisphere, and spatial distance). In total, 107 out of 154 connectivity matrices converged within this time window.

With the caveat that this does not represent the full sample, results examining the influence of topological parameters for individual matrices mirror our results at the aggregate level. Though there was individual variability, all participants had a significantly positive triads parameter and a significantly negative two-stars parameter (see Figure S1). This is thus further evidence that the default mode network, whether examined in aggregate or the individual level, appears to be demonstrating segregated highway structure.

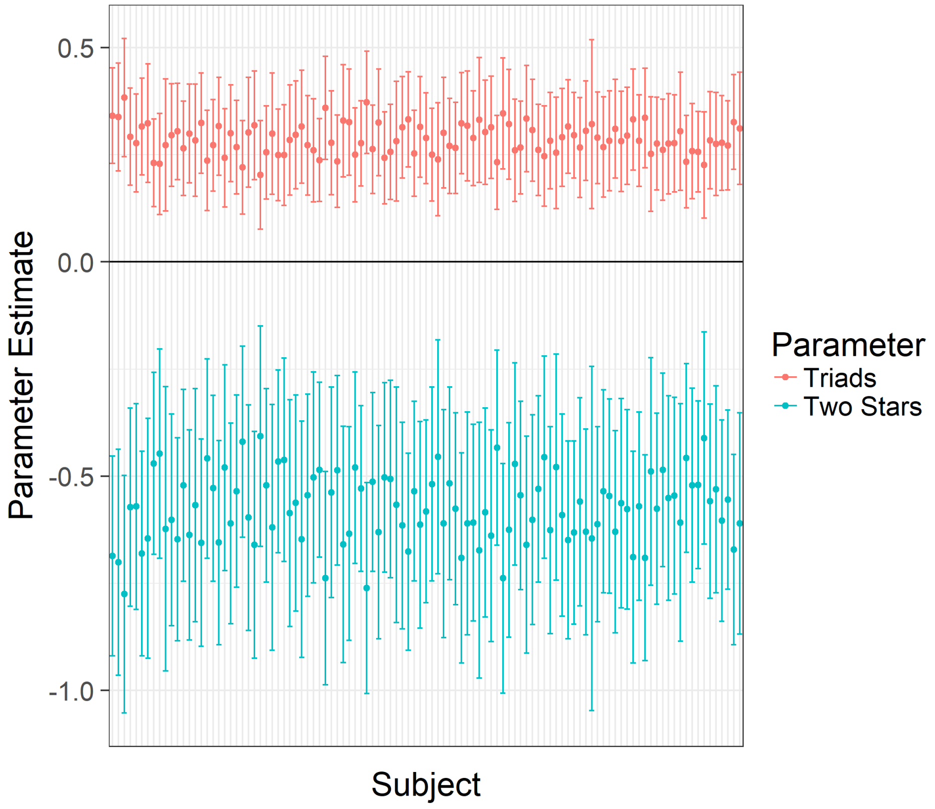

Figure S1: Parameter estimates for triads (red) and two-stars (blue) for every participant whose model converged. Error bars correspond to 95% confidence intervals.

Supplemental Results – GOF plots of all models

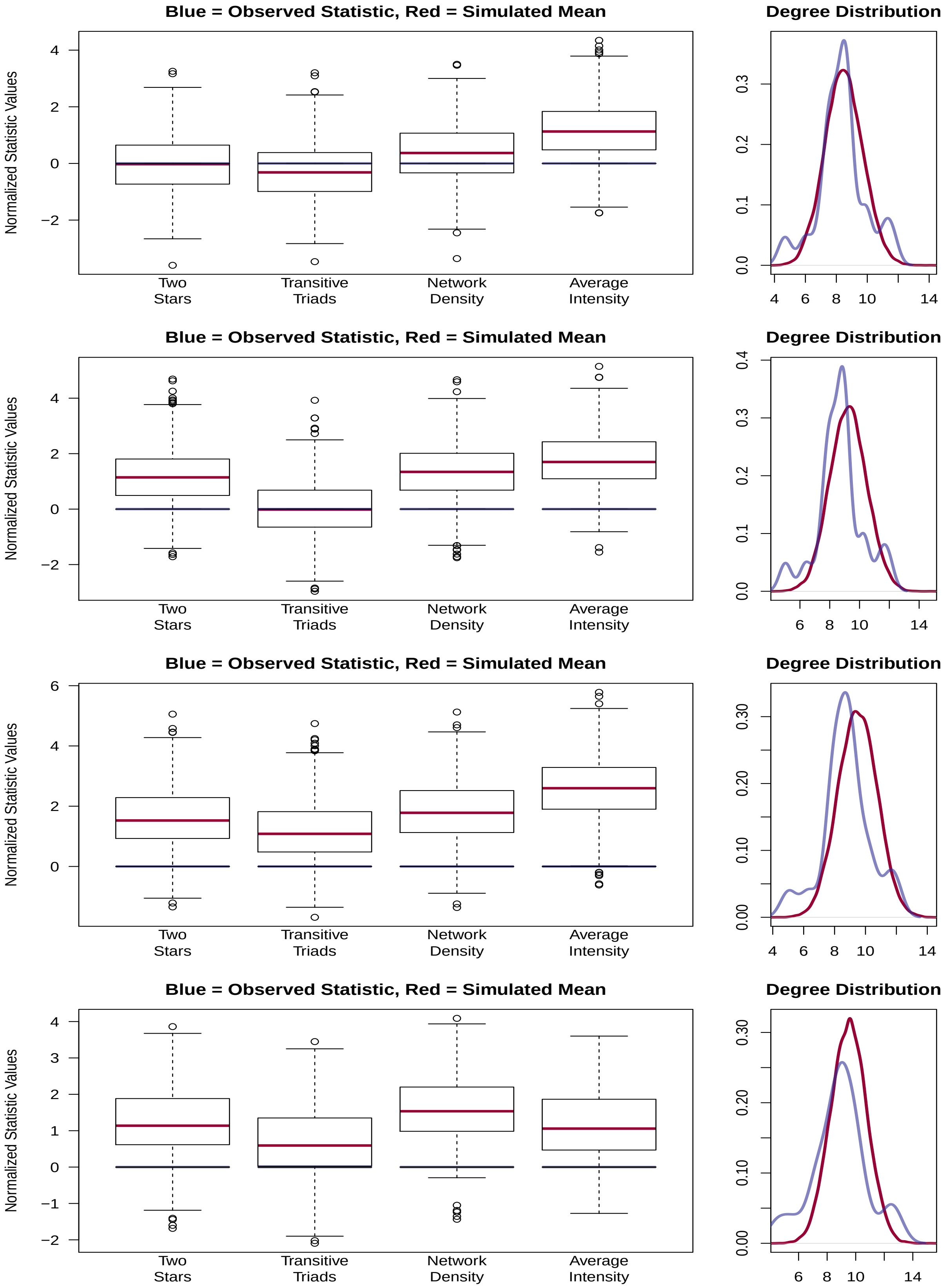

Figure S2: GOF plots for models with only an edges term and one other statistic. From top to bottom: two-stars, triads, hemisphere, and distance.

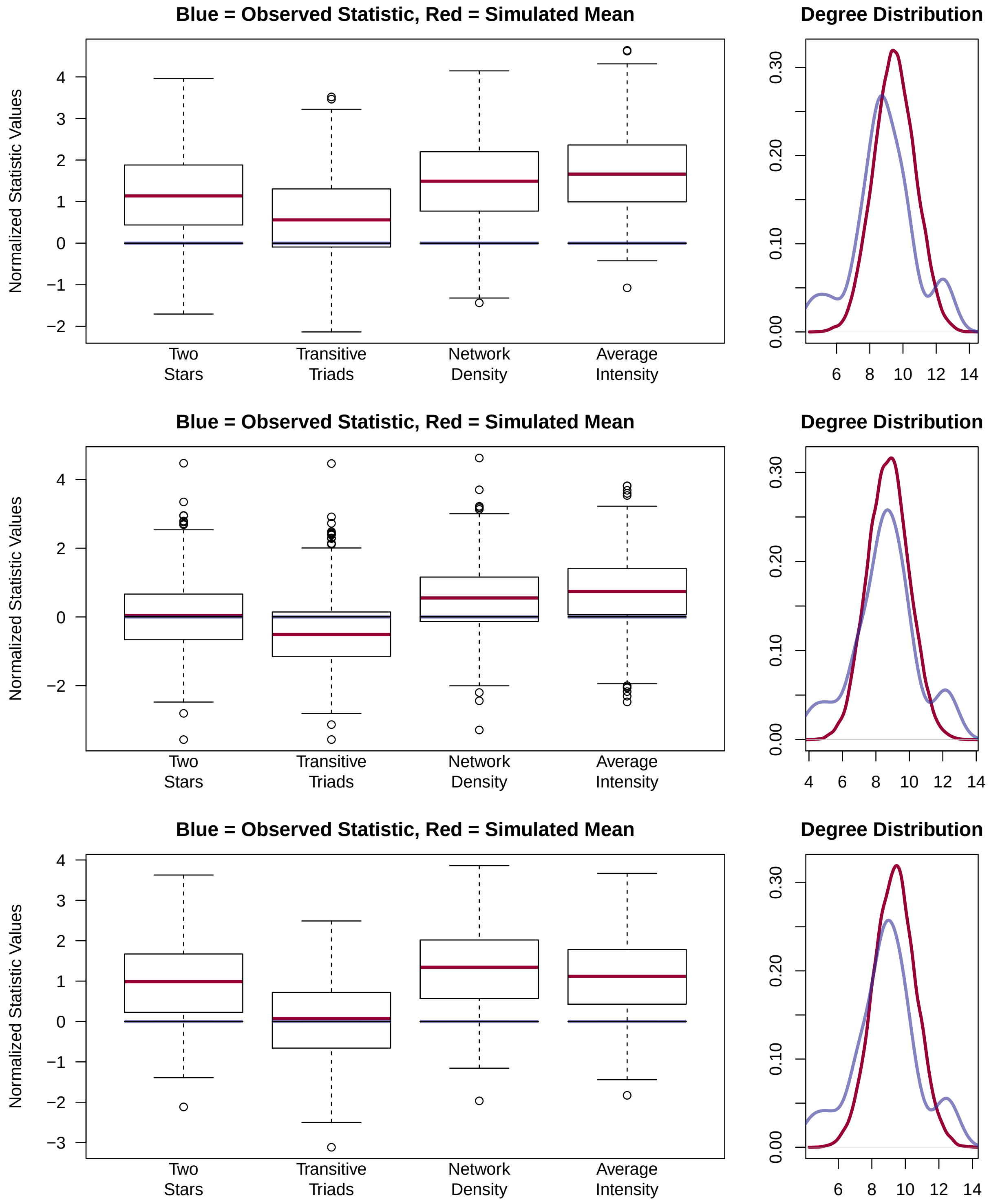

Figure S3: GOF plots for models with three statistics: (top) edges, distance, and hemisphere, (middle) edges, distance, and two-stars, (bottom) edges, distance, and triads.

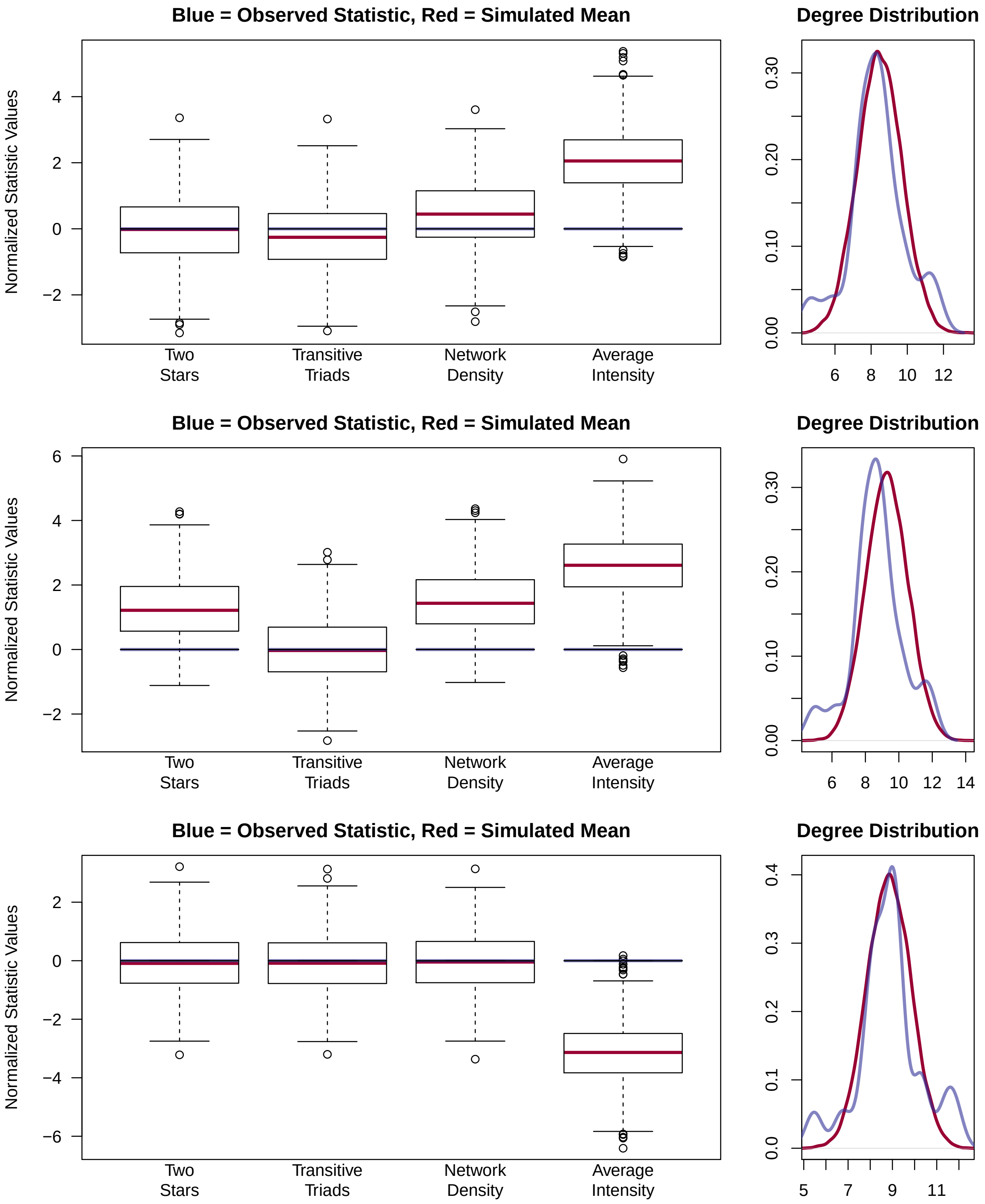

Figure S4: GOF plots for models with three statistics (continued): (top) edges, hemisphere, and two-stars (middle) edges, hemisphere, and triads, (bottom) edges, two-stars, and triads.

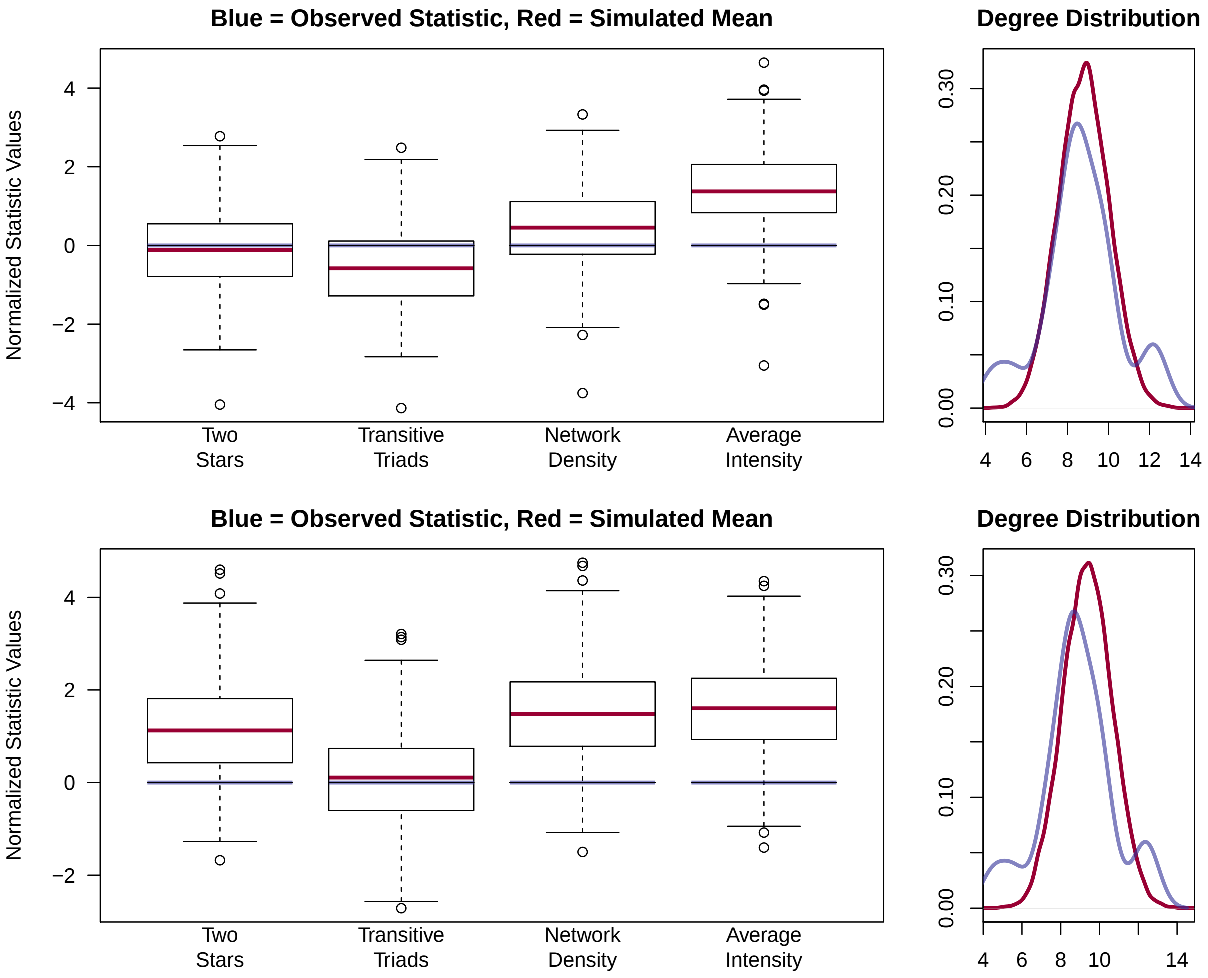

Figure S5: GOF plots for models with four statistics: (top) edges, hemisphere, distance and two-stars, (bottom) edges, hemisphere, distance, and triads.

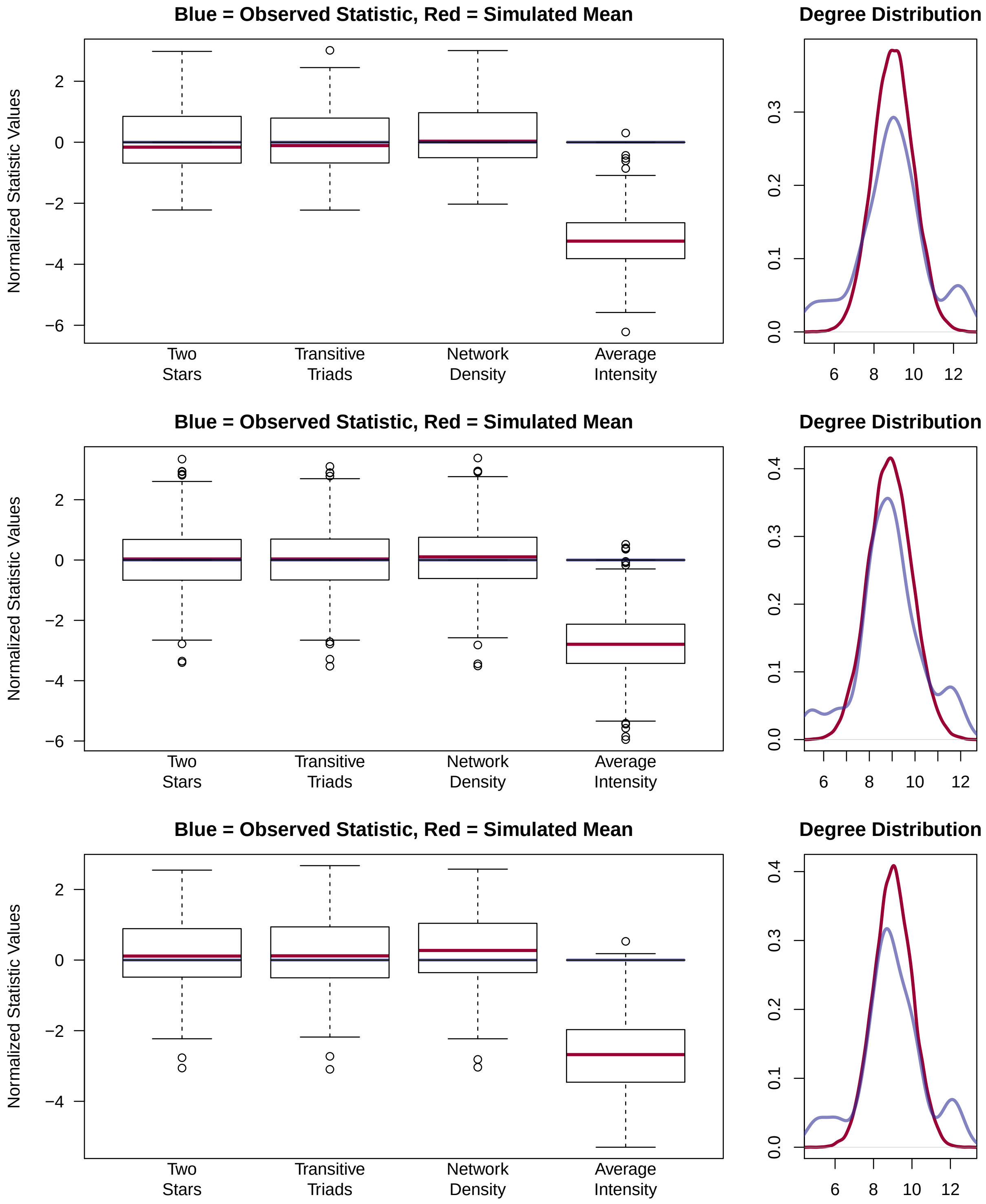

Figure S6: GOF plots for models: (top) edges, distance, two-stars, and triads, (middle) edges, hemisphere, two-stars, and triads, (bottom) all five statistics of edges, distance, hemisphere, two-stars, and triads.
